# The role of Endometriosis Clinical Nurse Specialists in British Society for Gynaecological Endoscopy registered centres: A UK survey of practice

**DOI:** 10.1002/nop2.574

**Published:** 2020-07-23

**Authors:** Wendy Norton, Helene Mitchell, Debra Holloway, Caroline Law

**Affiliations:** ^1^ Faculty of Health and Life Sciences De Montfort University Leicester UK; ^2^ McNair Centre Guys and St. Thomas' NHS Foundation Trust London UK

**Keywords:** clinical nurse specialist, commissioned services, endometriosis, nurses, nursing, role development

## Abstract

**Aim:**

To identify Endometriosis Nurse Specialists' roles and responsibilities in accredited Endocentres, how these align with the Royal College of Nursing Skills Framework and potential for development to improve patient care.

**Design:**

Cross‐sectional national survey.

**Methods:**

Data were collected from an online survey distributed to all 66 Endometriosis Nurse Specialists working across 58 UK‐based Endometriosis registered centres. The response rate was 58% (*N* = 38). Data from closed questions were analysed using descriptive statistics, and free text responses were collated and analysed thematically.

**Results:**

Unlike Nurse Specialists in other fields of practice, most Endometriosis Nurse Specialists (*N* = 33, 87%) had another nursing role. The median hours worked per week as an endometriosis nurse was only 13.5 hr. Most respondents (*N* = 35, 92%) spent all or most of their allocated hours collecting and inputting endometriosis data, whilst over a third (*N* = 13, 34%) were not undertaking any autonomous, nurse‐led patient consultations.

## 1 INTRODUCTION

Endometriosis is an oestrogen‐dependent chronic condition where endometrial tissue forms lesions outside the uterus, inducing a chronic, inflammatory reaction (Dunselman et al., 2014). Symptoms of endometriosis vary in their presentation and severity and include pelvic pain, painful periods, chronic fatigue, dyspareunia and infertility, which can have a significant physical, sexual, psychological and social impact on women's lives (National Institute for Health & Care Excellence [NICE], 2017). The true prevalence of endometriosis is difficult to establish due to its uncertain and enigmatic nature; however, it is estimated to affect between 2%–10% of women in the general population but up to 50% of infertile women (Eskenazi & Warner, 1997; Meuleman et al., 2009). A diagnosis of endometriosis can only be confirmed definitively following histology of a directly biopsied vaginal lesion, from a scar, or of tissue collected during laparoscopy (European Society of Human Reproduction & Embryology [ESHRE], 2013). The length of time from first symptom onset to surgical diagnosis is often complicated by women delaying seeking help, as they perceive their painful periods to be normal and misdiagnoses by healthcare professionals (Hudelist et al., 2012). This has resulted in an average delay in diagnosis of 5–8.9 years (Culley et al., 2013), during which time the disease is progressing, which may compromise the woman's fertility (Agarwal et al., 2019).

The cause of endometriosis is uncertain, and there is no definitive cure (World Endometriosis Society & the World Endometriosis Research Foundation, 2012). Endometriosis constitutes a significant burden on the quality of life of women and their families, as well as on healthcare systems, as a result of direct and indirect costs (Eisenberg, Weil, Chodick, & Shalev, 2018; Nnoaham et al., 2011), similar to other chronic diseases such as diabetes, Crohn's disease and rheumatoid arthritis (Simoens et al., 2012). The total annual societal burden of endometriosis‐associated symptoms for Europe has been estimated to be between 0.8–12.5 billion euros, based on average costs per woman treated in referral centres (Nnoaham et al., 2011; Simoens et al., 2012). Women with endometriosis have complex needs and require long‐term support (NICE, 2017). Nurses play a pivotal role in the delivery of high‐quality holistic care to people with long‐term conditions (Coulter, Roberts, & Dixon, 2013), yet to date there is a dearth of evidence on the nurse's role in supporting women with endometriosis.

## 2 BACKGROUND

In 2013, the NHS England Health Service Commissioning Board, in response to the European guidance on the management of women with endometriosis (ESHRE, 2013), specified that cases of severe endometriosis should be managed by dedicated specialist centres that have been accredited by the British Society for Gynaecological Endoscopy (BSGE). Severe endometriosis is defined as either deeply infiltrating endometriosis or rectovaginal endometriosis, which has an annual incidence of around 5,000 new cases in the UK per year (NHS England, 2013). Deeply infiltrating endometriosis exists where the disease invades at least 5 mm below the tissue surface and can occur in a variety of sites; this includes the bladder, pelvic sidewalls, ovaries, pelvic brim, bowel surface and diaphragm. Rectovaginal endometriosis is endometriosis that involves the rectovaginal septum area (rectovaginal septum; vagina; utero‐sacral ligaments; rectum) (NHS England, 2013). Removing the endometriosis in these cases involves complex surgery and can be associated with high rates of serious complications (Byrne et al., 2018). National BSGE criteria (see www.bsge.org.uk) now exist which set out the standards of service and workload required to undertake surgical excision of advanced endometriosis. To retain accreditation as a BSGE Endocentre, all consenting patients who undergo surgery for deep rectovaginal endometriosis that includes dissection of the pararectal space in an Endocentre must have their data entered on to the BSGE national endometriosis database (Byrne et al., 2018). This increasing caseload is driving the establishment of endometriosis centres where such work can be undertaken by specialist multidisciplinary teams (MDT).

The primary aim of endometriosis centres is to provide woman‐centred specialist care that helps improve the quality of life for women with severe endometriosis. In the NHS Commissioning Board Specification (NHS England, 2013), clear reference is made to the role of the Clinical Nurse Specialist (CNS) whose role is to liaise directly with women using the specialist service and provide women with support in the management of the condition. This is the first NHS England Health Service commission to stipulate the importance and accreditation, of a specialist centre being dependent on, having a CNS on the MDT. Nurse specialists have been described as highly skilled registered nurses who provide an optimal return on investment in terms of income generation, patient safety and efficiency, cost savings and improvements in patient care and experience (Royal College of Nursing [RCN], 2010). NHS England (2013) commissioning service specification outlines the key roles of the specialist nurse as: initial outpatient assessment in specialist clinics, information and counselling provision, completing primary Quality of Life questionnaires and undertaking elective outpatient follow‐ups at 6 months post‐surgery. The CNS is the interface between the patient and the specialist team.

**FIGURE 1:**
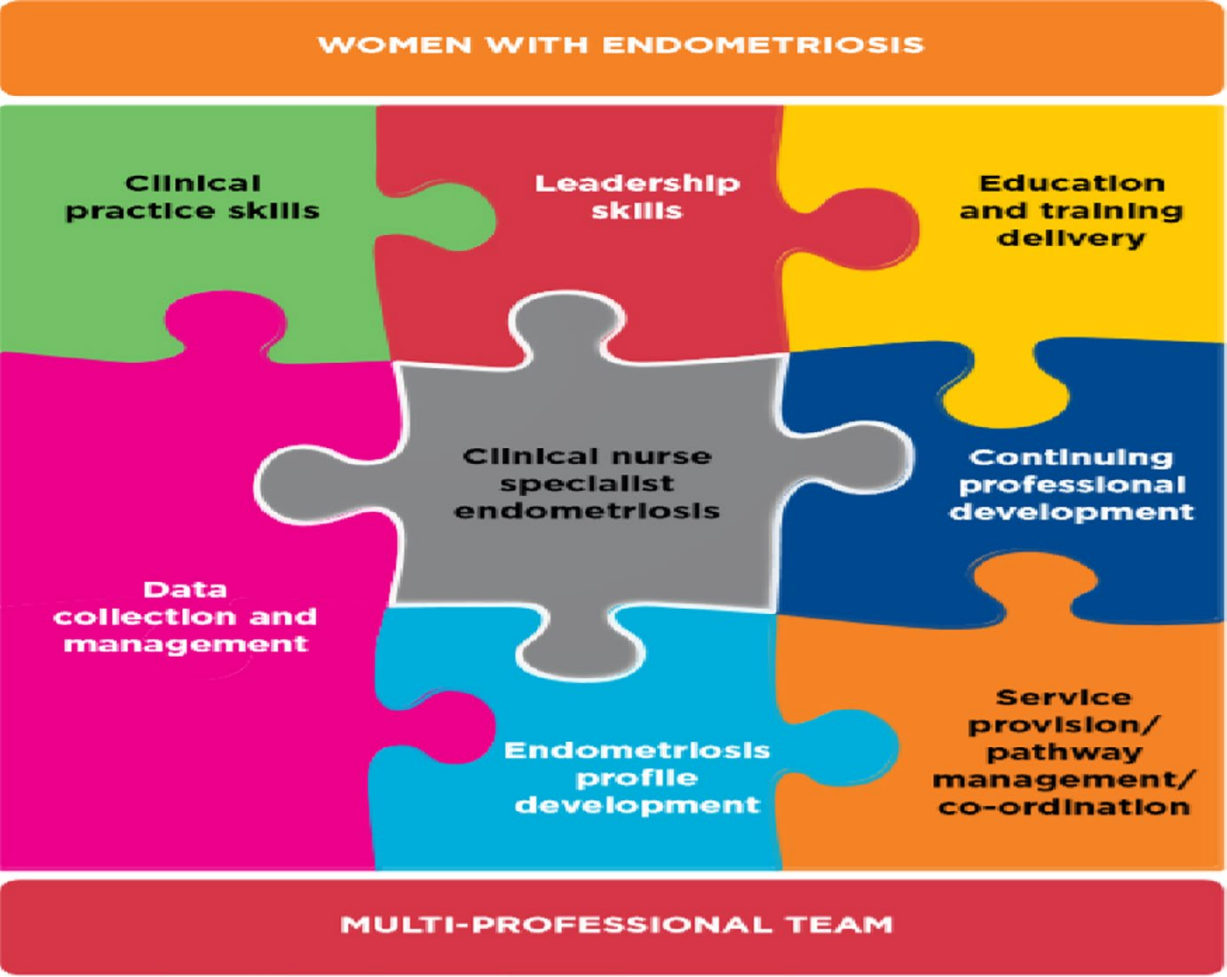
Overview of Clinical Nurse Specialist Skills (Jigsaw puzzle image reproduced with kind permission of the Royal College of Nursing)

As BSGE specialist centres expand and develop across the UK, the CNS workload is set to evolve. Recognizing the lack of a national standard to define this role, the RCN, in collaboration with Endometriosis UK, a national patient support charity and the BSGE, devised a skills and knowledge framework that would inform and enhance local practice and establish a baseline nursing standard across the UK (Royal College of Nursing, 2015). This framework defined the role and responsibilities of the Endometriosis CNS and provided a career pathway enabling nurses to further develop skills required for this role to ultimately improve care delivery. This CNS role is complex, demanding a range of clinical, management and leadership skills to be developed and an expectation that the CNS works as an autonomous practitioner (see Figure 1), as seen in other fields of clinical practice, such as oncology (National Cancer Action Team, 2010), diabetes (Riordan, McHugh, Murphy, Barrett, & Kearney, 2017), menopause (Royal College of Nursing, 2019) and fertility (Royal College of Nursing, 2018).

As the Endometriosis CNS role is yet to be evaluated, this survey was designed to identify Endometriosis CNSs' day‐to‐day roles and responsibilities, investigate how these align with the RCN skills framework and highlight areas requiring further development.

## 3 THE STUDY

### 3.1 Aim

The aim of this study was to identify Endometriosis CNS roles and responsibilities in BSGE registered centres, explore how these align with the RCN Skills Framework and establish potential for further development to improve care for women with endometriosis.

### 3.2 Design

This study employed a cross‐sectional descriptive design, using an online survey and adheres to the CHERRIES guidelines for e‐surveys.

### 3.3 Method

The sampling frame was developed from the endometriosis accredited centre information on the BSGE website, which listed the nurse specialist/s employed at each centre. Sixty‐six CNSs working in the 58 UK‐based BSGE centres (the number of UK‐based BSGE centres at the time of commencing this study) listed on the website (50 fully accredited centres and eight provisionally accredited centres) were invited to take part in the survey.

### 3.4 Data collection

Data were collected from 10th February 2017 to 31st March 2017. All CNSs listed on the BSGE website were sent an e‐mail from the BSGE Head Office containing a letter from the research team explaining the study and a link to the online survey, hosted by Qualtrics (www.qualtrics.com), a secure online survey provider (see Appendix S1).

Participants were able to partially complete the survey and return to it at any time whilst it remained available. Due to an initially slow response rate, the survey timeframe was extended from 4 weeks to seven and reminder e‐mails were sent from the BSGE Head Office to all CNSs at 4 weeks and just before the survey closed.

### 3.5 Ethics

This survey was approved by the local university Research Ethics Committee (Ref: 1903) on 31st January 2017. The introductory information stated that completion of the survey would confirm that the respondents had understood the purpose of the survey and consented to the use of the data as specified. Contact information for the study team was also provided to answer any queries from potential participants. Participation was anonymous to protect data confidentiality.

### 3.6 Analysis

Data were exported into SPSS (version 22) for analysis. Only submitted questionnaires were included in the final data set. Missing responses were coded and included in the analysis, and no categories were collapsed. Data from the closed questions were analysed using descriptive statistics, and free text responses were collated and analysed thematically and expressed as frequencies and proportions. Median values are reported for non‐normally distributed data. All data were analysed anonymously.

### 3.7 Rigour

The survey was designed to capture demographic information on endometriosis CNSs (e.g. duration and hours undertaking this role, UK pay scale banding, previous nursing experience and educational qualifications) and role and responsibilities based on the nurse specialist role domains (Royal College of Nursing, 2010) that formed the basis of the RCN Endometriosis CNS Skills Framework (Royal College of Nursing, 2015) as illustrated in Figure 1. Questions were asked about: clinical practice skills, service provision and pathway management (including leadership skills), education, training delivery and endometriosis profile development (including continuing professional development) and data collection and management. Further questions were designed to identify aspects of the role that CNSs felt were working well and those that remained challenging, CNSs' views on further development of their role and the skills they identified as most important for their on‐going professional development.

A range of response options was used to reflect the different questions, including a 0–5‐point Likert scale, yes/no responses and free text boxes. To establish content validity, a draft of the survey was reviewed by the BSGE, then piloted with a gynaecology nurse with previous clinical endometriosis experience. This process verified the acceptability of content, clarity of items, survey length and question format to nurses. Minor revisions were made to three questions for clarity prior to distribution. The final survey questionnaire was approved by BSGE officers prior to dissemination.

## 4 RESULTS

Of the initial 66 endometriosis CNSs who were invited to take part in the survey, 38 completed the survey, producing a response rate of 58%. Some questions allowed for more than one response and not all respondents answered all questions, consequently data may not equate to the number of respondents.

### 4.1 Demographic characteristics

The respondents had an average of 21.85 years' post‐registration clinical experience (CI 18.98–24.72, range 4.5–35 years) and had worked specifically in women's healthcare for an average of 15.88 years (CI 13.18–18.57, range 1.25–30 years). Of note is that five respondents had not undertaken any women's health‐related courses or training. The RCN Skills Framework highlights that CNSs should ideally be working towards Masters level thinking and problem‐solving, yet only 11 (29%) of the respondents were educated to Master's level.

When respondents were asked how they came to be an Endometriosis CNS, 14 had applied for this specific post, six respondents had taken on this role as an internal secondment; in two cases, the endometriosis CNS job was advertised as a joint post with their current role, and in one case, the respondent had been personally asked to apply for the role. However, of note is that in 15 cases (39%), the endometriosis CNS role had just been added to a nurse's current gynaecology‐related role rather than them having applied for the specific post.

Most participants (*N* = 33, 87%) had at least one other nursing role alongside their Endometriosis CNS role and of these participants, 27 (82%) had one other role, four participants had two other nursing roles and two participants had three other nursing roles. These other roles related to the following: gynaecology assessment/Advanced Nurse Practitioner (*N* = 9); gynaecology clinic/ward management (*N* = 10); surgical practitioner/theatres (*N* = 3); hysteroscopy/colposcopy practitioners (*N* = 3); fertility care (*N* = 2); gynaecology oncology (*N* = 2); early pregnancy care (*N* = 2); ultrasonography (*N* = 2). The hours worked in these other roles ranged from 4 hr–37.5 hr indicating that some CNSs were working more than their contracted weekly hours to keep up with their caseload.

There was a wide variation in the hours allocated to undertake the endometriosis CNS role, ranging from just 5 hr per week to the maximum full‐time 37.5 hr per week. The median hours worked per week as an Endometriosis CNS was 13.5 hr. On a positive note, 26 (68%) of the respondents did report that their endometriosis CNS hours were protected; this may relate to the required 12 complex cases each gynaecological surgeon must perform annually to maintain a centre's accreditation (British Society for Gynaecological Endoscopy, 2017).

Respondents had been employed in the Endometriosis CNS role for a mean of just 18 months with a range of 1 month to the maximum 48‐month timeframe since the commissioning of this role in 2013. Differences in the UK pay scale banding for the CNS role were reported, with three respondents being paid at band eight, 19 respondents at band seven, 14 respondents at band six and one respondent only being paid at band five, which is lower than the minimum pay band six awarded to CNSs in other disciplines (NHS Careers, 2011).

### 4.2 Clinical practice skills

Participants were asked about the clinical skills they had gained during their career and whether these skills were being used within their Endometriosis CNS role, as outlined in the RCN Skills Framework. Table 1 indicates the clinical practice skills of those who answered these questions and responses varied across items. Some respondents with specific skills prior to becoming an Endometriosis CNS appeared not to be using these skills within the CNS role, which may potentially lead to them becoming de‐skilled in technical aspects of clinical practice. The biggest decrease in skill use relates to undertaking ultrasound scanning and working to patient group directions (PGDs). In contrast, there was a reported increase in the use of some skills, such as educating women on medical and complementary therapy options.

**Table 1:** Clinical practice skills

	Did you have this skill before becoming an endometriosis CNS?		Do you currently use this skill within your endometriosis CNS role?	
	Yes (%)	No (%)	Yes (%)	No (%)
Non‐medical prescriber	9 (23.7)	28 (73.7)	7 (18.4)	25 (65.8)
Work to patient group directions (PGDs)	12 (31.6)	23 (60.5)	4 (10.5)	26 (68.4)
Order patient investigations	26 (68.4)	12 (31.6)	22 (57.9)	11 (28.9)
Take consent for medical interventions	9 (23.7)	28 (73.7)	10 (26.3)	23 (60.5)
Undertake ultrasound scanning	7 (18.4)	30 (78.9)	2 (5.3)	31 (81.6)
Undertake physical examinations	14 (36.8)	23 (60.5)	12 (31.6)	20 (52.6)
Undertake patient counselling/psychological support	30 (78.9)	7 (18.4)	31 (81.6)	2 (5.3)
Make referrals to other specialist teams	22 (57.9)	14 (36.8)	24 (63.2)	7 (18.4)
Provide women with information on medical treatment options	26 (68.4)	11 (28.9)	29 (76.3)	3 (7.9)
Educate women on complementary therapy options	16 (42.1)	21 (55.3)	23 (60.5)	10 (26.3)
Undertake audits	24 (63.2)	13 (34.2)	21 (65.6)	11 (34.4)
Undertake research	11 (28.9)	26 (68.4)	9 (23.7)	21 (55.3)
Help recruit patients to research studies	18 (47.4)	19 (50.0)	15 (39.5)	17 (44.7)

### 4.3 Service provision, pathway management and leadership

A key aspect of the RCN skills framework related to the CNS as an autonomous practitioner, who undertakes consultations independently whilst also working across and within multiple specialist teams. However, as outlined in Table 2, only eight (32%) respondents delivered nurse‐led face‐to‐face consultations, undertaking a median of five consultations per week, with a range of two to 12 consultations. Only a third of the respondents (*N* = 8, 33%) were undertaking nurse‐led telephone consultations, six CNSs (24%) were dealing with e‐mail enquiries, whilst 11 (58%) were managing more informal patient telephone enquiries. The median number of consultations ranged from 4–5 per week across all the consultation/ enquiry types. In addition, respondents identified a lack of opportunity to undertake or develop their leadership skills to support an autonomous practitioner role. Only three (8%) respondents reported spending their CNS allocated hours using and applying their leadership skills.

**Table 2:** Independent nurse‐led consultations: Numbers and types

Consultation/enquiry types	Responses	No. reporting zero	Median no. of consultations per week	Range minimum	Range maximum
Independent nurse‐led consultations	25	17 (68%)	5 (*n = *8)	2	12
Formal nurse‐led telephone consultations	24	16 (67%)	4 (*n* = 8)	1	10
Informal nurse‐led telephone enquiries	19	8 (42%)	5 (*n* = 11)	2	25
Nurse‐led e‐mail consultations	25	19 (76%)	5 (*n* = 6)	1	10

### 4.4 Education, training and profile development

The remit of the CNS also includes educational and profile development components. However, the findings from this study show that most CNSs were not involved in any endometriosis educational provision for healthcare professionals or patients and their partners. Only 15 (39%) CNSs were providing education for healthcare providers in secondary care, whilst only 8 (21%) were providing education for healthcare providers in primary care settings. Education sessions for women with endometriosis were provided by 16 (42%) CNSs, but only 10 (26%) offered any educational sessions to women's partners. In relation to profile development, only 11 (29%) respondents were involved with their local endometriosis support group and only three respondents had been involved with any independent research within their Endometriosis CNS role. With respect to the CNSs’ continuous professional development, most respondents (*N* = 27, 71%) had attended a BSGE annual meeting within the previous 2 years, whilst 23 (61%) reported not having any access to supervision, mentoring or buddying support for their role.

### 4.5 Data collection and management

The CNS allocated hours were not always aligned to patient contact. Endometriosis CNSs carried the burden of the administration work for the Endocentre with 35 (92%) participants reporting being responsible for collecting and inputting endometriosis data. This activity took up most of the participants' allocated CNS time and there were conflicting views on how well this aspect of the role was working, with seven CNSs reporting that this aspect of their role was working well, as opposed to 13 respondents who felt this aspect of the role remained challenging. The key concerns appeared to relate to the time required to chase the Quality of Life questionnaires, contacting patients to obtain accurate information for inputting and general training and database access issues that help maintain the Endocentre accreditation.

### 4.6 Aspects of the role that are working well and those remaining challenging

All CNSs were asked to comment on the aspects of the role they felt were working well and those they felt remained challenging. Qualitative comments from the respondents were analysed thematically and grouped into distinct areas.

The key aspect of the role that respondents perceived to be working well related to their direct contact with patients and continuity of patient care (*N* = 21), with 35 respondents (*N* = 92%) believing that their CNS role enabled them to be a patient advocate. Other positive aspects of the role were reported to be the multidisciplinary team approach (*N* = 15), nurse‐led clinics in centres where these were standard practice (*N* = 9), database management (*N* = 7), management support (*N* = 2) and in situations where CNSs were able to use their expert knowledge to support others (*N* = 2), assist in theatre cases (*N* = 2) and develop a new service (*N* = 2).

With regard to aspects of the role that remained challenging, respondents reported a general lack of allocated time to enable them to embrace core components of the CNS role (*N* = 12), data management as the priority for CNS hours (*N* = 12) and limited patient contact time (*N* = 8) as the main barriers to providing care. Other reported challenges were the NHS appointment, referral and operation waiting times and processes (*N* = 6), having to balance the endometriosis CNS role with another role (*N* = 4), the reluctance of medical staff to acknowledge the CNS role (*N* = 3) and lack of opportunities to undertake additional education to develop the role or service (*N* = 3) or educate others about endometriosis (*N* = 2).

### 4.7 Suggestions for role development

All CNSs were asked to comment on how they would like to further develop their role. Thirty‐one CNSs responded with suggestions (see Table 3). The most frequent suggestions related to developing the role as an autonomous practitioner, by undertaking more nurse‐led consultations and/ or clinics and being involved in more educational and patient advocacy‐related activities, both core elements of a CNS role.

**Table 3:** Future role development

What CNSs would like to introduce or further develop within their role	Frequency
Develop nurse‐led clinics	10
Support group development/involvement	10
Develop advanced nurse practitioner skills	4
See pre/post‐operative patients	3
Raise endometriosis profile in primary care	3
Become a nurse prescriber	3
Develop endometriosis specific patient information	2
Dedicated admin/database support	2
Develop a counselling service	1
CNS job share to ensure a CNS is always available for patients	1
Sexual health education	1
Develop the new service	1

## 5 DISCUSSION

The most striking features from this survey are the significant variation in allocated CNS role hours, how these hours are used across BSGE centres and the Endometriosis CNS being a second role for 87% of the respondents. The lack of time dedicated to this CNS role, alongside the demands of their other roles, makes it impossible for CNSs to fulfil all of the RCN role domains. There was a lack of consensus amongst the respondents on the role priorities for the Endometriosis CNS hours, but precedence appears to be dictated by commissioning requirements, interpreted as recording and inputting surgical data to maintain the centre's accreditation. The RCN Skills Framework (Royal College of Nursing, 2015) aimed to establish a baseline practice standard for the Endometriosis CNS role to promote equity for women and their partners. However, the survey findings suggest that only a minority of CNSs are working at the recommended level of practice, have their own patient workload and are able to work autonomously. Given the limited role hours allocated, it is unsurprising that for many CNSs, core components of the role are unfulfilled, such as education, creating patient support groups, raising the profile of endometriosis, developing leadership skills to enhance practice and exercising levels of autonomy.

Whilst the CNS roles in other fields of practice, such as oncology, are well‐established full‐time roles that have developed over time in response to patient need (Leary, Mak, & Trevatt, 2011), the Endometriosis CNS role was imposed by healthcare commissioning in 2013 and is still in its infancy. Further work is needed to address the disparities in Endometriosis CNS hours, the clinical priorities for these hours and reassess the rationale for combining the Endometriosis CNS with other clinical roles, especially given the level of psychological support fertility and oncology patients, for instance, require.

Clinical Nurse Specialist roles are often poorly understood or valued (Royal College of Nursing, 2010). More work is needed to increase clinicians' and managers' understanding of the diverse and valuable skills that CNSs bring to the team, the vital role they play in delivering high‐quality care and the economic contribution CNSs' autonomous practice can make to care provision. A CNS is a supportive resource who should act as the interface between patients and the specialist team and thus improve patient outcomes and free up consultant and clinic time. NHS England (2013) identify that the role of the specialist nurse is to carry out primary patient contact to triage referrals, complete Quality of Life questionnaires and undertake selected patient follow‐up consultations. Whilst the findings suggest that many of the CNSs had the advanced clinical skills required for the role, the median 13.5 hr per week role allocation, mostly spent on data collection and management, greatly restricts the number of patients the CNS is able to support. Data collection is key to patient assessment, but a distinction needs to be made between Quality of Life data collection for implementing and evaluating patients' care plans and generic data inputting to maintain the Endocentre's accreditation. General administration work and data inputting could be undertaken by an administrator, freeing up time for CNSs to provide more patient‐facing care and interventions in line with the NHS England (2013) commissioning remit.

The commissioning definition of *severe endometriosis* which only applies after a woman has undergone surgery results in a two‐tiered classification (severe and non‐severe endometriosis), limiting who has access to a nurse specialist. In reality, CNSs also care for women with less severe endometriosis, as well as those with severe endometriosis who have not undergone surgery, in non‐specialist gynaecology services. The National Institute for Health and Care Excellence (2017) has recognized the equal need for specialist nurse support for these additional patient cohorts. However, currently, this caseload is not counted in the CNS workload because these patients fall outside of the commissioning specification that generated this role. The CNS role hours should be proportionate to the patient population and women's needs, as with the Breast Screening CNS role (Public Health England, 2019) and reviewed annually to accurately reflect the current patient caseload, as BSGE centres monitor women who have had surgery for endometriosis for at least 2 years. Women who may benefit from contact with an Endometriosis CNS could also be identified through a Holistic Needs Assessment completed at diagnosis, during or after treatment, as undertaken by oncology CNSs (Snowden et al., 2014). Endometriosis CNS hours would then directly reflect women's needs, rather than being task‐focused which appears to be the current situation.

The benefits of autonomous practice are evident from other CNS and advanced nursing roles, for example, hysteroscopy, fertility and early pregnancy, where nurses manage their own caseload of complex patients (Royal College of Nursing, 2009). CNSs need to develop their clinical and leadership skills through further education and training, personal reflection and buddying/mentoring from more experienced CNSs so they can effectively lead improvements in endometriosis care provision. The RCN Skills Framework could be incorporated into BSGE practice standards to embed core aspects of the role in service provision and pathway management. Endometriosis CNSs could annually evaluate and report the role domains they have developed, the number of autonomous clinics undertaken and related costs in terms of freed up clinical or clinician time.

Continuous expansion of BSGE centres presents an opportunity for CNSs to develop effective leadership to enhance women's care. We propose developing bespoke leadership masterclasses to enable nurses to tap into their clinical expertise, promote understanding of resource management and business planning and strengthen their pivotal role; all valuable skills needed to take the role forward. In turn, demonstrating the value the CNS brings to the MDT can act as a catalyst for increasing the hours allocated to the role across BSGE centres.

### 5.1 Strengths and limitations

This is the first study to explore how the commissioned Endometriosis CNS role is operationalized in BSGE registered centres, the extent to which their roles and responsibilities align with the Royal College of Nursing, (2015) skills framework and to identify priorities for future role development. However, the findings should be viewed in light of the following limitations: firstly, the sample size was small, reflecting the number of CNSs in post at the time of the survey; whilst the number of Endometriosis CNSs is now increasing, this is still a limited cohort. Secondly, this is a self‐selecting sample; other endometriosis CNSs who did not take part in the survey may be undertaking roles aligned to the RCN skills framework and be working as autonomous practitioners. Given the increasing number of BSGE accredited centres and subsequent increase in Endometriosis CNSs, a follow‐up survey to assess the CNS evolving role is warranted.

## 6 CONCLUSION

The Endometriosis CNS role has been driven by commissioning services, identifying a gap in service provision, which appears to follow a medical perspective rather than a holistic nursing approach to person‐centred care. This emphasis has led to a specific angled focus on how this role has developed, rather than being guided by patients' needs. As the number of accredited BSGE Endocentres and thus the number of Endometriosis nurses increases, there is an urgent need to review the CNS allocated hours to create an effective uniformed provision across BSGE registered centres. We conclude that there is a real opportunity for CNSs to take ownership for this role development enabling them to more effectively respond and adapt to patients' emerging needs. In addition, further research is needed to assess the cost benefits of Endometriosis nurse specialist‐led activities, including nurse‐led clinics, to inform local workforce and service improvement strategies.

By working in conjunction with patients and patient support groups and learning from CNSs in other fields of practice and in other jurisdictions, Endometriosis CNSs can reflect on the pivotal role they play within MDTs and identify how they can advance this role to enhance person‐centred care. The findings from the present study have informed the development of a bespoke endometriosis CNS leadership masterclass designed to help CNSs demonstrate the value of nurse‐led innovations and services and promote effective CNS deployment to drive role development, reduce inefficiency and enhance the quality of care for women with endometriosis across the UK.

## CONFLICT OF INTEREST

None.

## AUTHORS CONTRIBUTION

WN, HM, DH and CL: Conception and planning of the study and contribution to the writing of the manuscript. WN, HM and DH: Survey questionnaire, collection of data and analyses.

## Supporting information

App S1
