# Supplementary material for: The role of Endometriosis Clinical Nurse Specialists in British Society for Gynaecological Endoscopy registered centres: A UK survey of practice
Source: Nurs Open. 2020 Jul 23;7(6):1852–60. doi: 10.1002/nop2.574 (PMC7544879; doi:10.1002/nop2.574)
Supplement: Supplementary file 1 — App S1 [file NOP2-7-1852-s001.docx]

Appendix 1. Checklist for Reporting results of Internet E-Surveys (CHERRIES)*

| **Item Category** | **Checklist Item** | **Page No.** | **Description** |
| --- | --- | --- | --- |
| Design | Study Design | Page 7. | The target population was endometriosis Clinical Nurse specialists (CNS) working in UK-based British Society for Gynaecological Endoscopy (BSGE) registered centres Endocentres. The sampling frame was developed from the endometriosis accredited centre information on the BSGE website, which listed the nurse specialist/s employed at each of the 58 BSGE centres listed on the website (50 fully accredited centres and eight provisionally accredited centres). |
| Ethics | Ethics approval | Page 8 and Supplementary File 1 | Ethics approval was obtained from the De Montfort University, Health and life sciences Faculty Research Ethics Committee (Ref: 1903) on 31^st^ January 2017. |
|  | Informed Consent |  | All participants were sent an introductory information email stating that the survey included questions about their current role, that it would take about 20-25 minutes to complete, that all responses were confidential and anonymous, and the data from all endometriosis CNSs would be collated and reported anonymously, individual nurses would not be identified in reporting, and their participation would not be divulged to any third parties. Consent was indicated by respondents clicking on the ‘SUBMIT” button to submit their answers. Respondents were told that once they had done this, they would not be able to withdraw their information. |
|  | Data protection | Page 7 and Supplementary File 1 | Propriety survey software and local servers were used to ensure data protection. No personal information was linked to the survey results. The fully de-identified dataset is kept on password protected computers. |
| Development and pre-testing |  | Pages 8 - 9 and Supplementary File 1 | The survey instrument was designed using the roles and responsibilities based on the nurse specialist role domains (RCN, 2010) that formed the basis of the RCN Endometriosis CNS Skills Framework (RCN, 2015), i.e. clinical practice skills, service provision and pathway management, education, training delivery and endometriosis profile development, and data collection and management. Further questions were designed to identify aspects of the role that CNSs felt were working well and those that remained challenging, CNSs’ views on further development of their role, and the skills they identified as most important for their on-going professional development. A draft of the survey was reviewed by the BSGE, then piloted with a gynaecology nurse with previous clinical endometriosis experience for ambiguity and clarity, acceptability to the participants, and validity. Minor revisions were made to three questions for clarity prior to distribution. The final survey questionnaire was approved by BSGE officers prior to dissemination. |
| Recruitment process | Open vs closed survey | Page 7 and Supplementary File 1 | This was a closed survey, only open to the CNSs who had been identified as working at each of the 58 BSGE centres listed on the BSGE website (as described above). |
|  | Contact Mode |  | The initial survey information and link, and an email reminder to follow-up on non-responses were distributed to all CNSs via the BSGE administrator, who held all the contact email addresses. |
|  | Advertising the survey |  | The survey was not advertised. Purposively selected nurses working as endometriosis CNSs in BSGE registered centres were invited to participate via the method described above. |
| Survey administration | Web/email | Pages 7-9 and Supplementary File 1 | This is an online survey, with respondents sent an e-mail from the BSGE Head Office containing a letter from the research team explaining the study, and a link to the online survey, hosted by Qualtrics ([www.qualtrics.com](http://www.qualtrics.com)). Reponses were collected through this secure online survey platform and stored on secure local servers. A range of response options was used to reflect the different questions, including a 0-5-point Likert scale, yes/no responses, and free text boxes. |
|  | Context |  | The survey was hosted by Qualtrics ([www.qualtrics.com](http://www.qualtrics.com)), a secure online survey provider. |
|  | Mandatory / voluntary |  | Voluntary survey. |
|  | incentives |  | No incentives were offered. |
|  | Time/date |  | Responses were collected between 10^th^ February 2017 to the end of March 2017. |
|  | Item randomisation |  | No randomisation of items was used. |
|  | Adaptive questioning |  | Relevant survey items were displayed based on previous responses (e.g. in five questions, respondents were able to skip questions if they had answered “No”. |
|  | Number of items |  | The full survey contained 75 items, spilt across four sections of questions. Not all respondents answered all items because of the adaptive nature of the questionnaire. |
|  | Number of screens |  | The full survey was distributed over approximately 10 pages. |
|  | Completeness check |  | Respondents were prompted to complete outstanding responses, but could proceed if they chose not to. |
|  | Review step |  | Respondents were unable to change their responses once submitted. The survey information sheet clearly stated that “*At the end of the survey you will be asked to confirm you are happy for your data to be submitted. This gives you the option to withdraw from the survey and none of your responses will be collected. However, once you submit the survey it will not be possible to withdraw, as the questionnaires are completed anonymously so it will not be possible to identify your specific information”.* |
| Response rates | Unique site visitor | Page 9 | No |
|  | View rate |  | No |
|  | Participation rate |  | 59 |
|  | Completion rate |  | 38 |
| Preventing multiple entries from same individual | Cookies used |  | No |
|  | IP check |  | No |
|  | Log file analysis |  | Not used. |
|  | Registration | Page 7 and Supplementary File 1 | Entry to the survey was via a unique link provided to each invitee to the survey. |
| Analysis | Handling of incomplete questionnaires | Page 8. | Only submitted questionnaires were included in the final dataset. |
|  | Questionnaires with atypical timestamp |  | Participants were able to go back to the survey so a cut-off point was not used for the timeframe. |
|  | Statistical correction |  | No weighting used. |

* Eysenbach, G. Improving the quality of Web surveys: the Checklist for Reporting Results of Internet E-Surveys (CHERRIES). J. Med Internet Res 2004; 6:e34.
